# *Anopheles gambiae* populations from Burkina Faso show minimal delayed mortality after exposure to insecticide-treated nets

**DOI:** 10.1186/s13071-019-3872-2

**Published:** 2020-01-10

**Authors:** Angela Hughes, Natalie Lissenden, Mafalda Viana, Kobié Hyacinthe Toé, Hilary Ranson

**Affiliations:** 1 Department of Vector Biology, Liverpool School of Tropical Medicine, Liverpool, L3 5QA UK; 2 Institute of Biodiversity, Animal Health and Comparative Medicine, College of Medical, Veterinary and Life Sciences, University of Glasgow, Glasgow, G12 8QQ UK; 3 Centre National de Recherche et de Formation sur le Paludisme, 01 BP 2208 Ouagadougou 01, Burkina Faso

**Keywords:** Mosquito, *Anopheles*, Insecticide resistance, Delayed mortality, Longevity, Sub-lethal effects, Long-lasting insecticidal nets (LLINs), Burkina Faso

## Abstract

**Background:**

The efficacy of long-lasting insecticidal nets (LLINs) in preventing malaria in Africa is threatened by insecticide resistance. Bioassays assessing 24-hour mortality post-LLIN exposure have established that resistance to the concentration of pyrethroids used in LLINs is widespread. However, although mosquitoes may no longer be rapidly killed by LLIN exposure, a delayed mortality effect has been shown to reduce the transmission potential of mosquitoes exposed to nets. This has been postulated to partially explain the continued efficacy of LLINs against pyrethroid-resistant populations. Burkina Faso is one of a number of countries with very high malaria burdens and pyrethroid-resistant vectors, where progress in controlling this disease has stagnated. We measured the impact of LLIN exposure on mosquito longevity in an area of the country with intense pyrethroid resistance to establish whether pyrethroid exposure was still shortening mosquito lifespan in this setting.

**Methods:**

We quantified the immediate and delayed mortality effects of LLIN exposure using standard laboratory WHO cone tests, tube bioassays and experimental hut trials on *Anopheles gambiae* populations originating from the Cascades region of Burkina Faso using survival analysis and a Bayesian state-space model.

**Results:**

Following single and multiple exposures to a PermaNet 2.0 LLIN only one of the four mosquito populations tested showed evidence of delayed mortality. No delayed mortality was seen in experimental hut studies using LLINs. A delayed mortality effect was only observed in WHO tube bioassays when deltamethrin concentration was increased above the standard diagnostic dose.

**Conclusions:**

As mosquito pyrethroid-resistance increases in intensity, delayed effects from LLIN exposure are substantially reduced or absent. Given the rapid increase in resistance occurring in malaria vectors across Africa it is important to determine whether the failure of LLINs to shorten mosquito lifespan is now a widespread phenomenon as this will have important implications for the future of this pivotal malaria control tool.
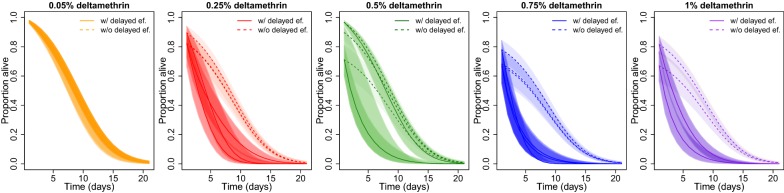

## Background

Long-lasting insecticidal nets (LLINs), which are the mainstay of many malaria control programmes in Africa, reduce contact between mosquitoes and humans by providing both a physical barrier and an insecticidal effect [1, 2]. In areas where LLINs are used on a large scale, they provide both personal and community-wide protection [3–5]. Across sub-Saharan Africa, ever-increasing numbers of people at risk of malaria are sleeping under an LLIN and this has been attributed to averting approximately two-thirds of potential malaria cases between 2000 and 2015 [6]. In Burkina Faso, malaria transmission remains high, and cases are increasing [7] despite high coverage of vector control tools, including three national LLIN distribution campaigns in 2010, 2013 and 2016. The majority of distributed LLINs were pyrethroid only, predominately deltamethrin; however, a small number of alphacypermethrin nets and nets containing piperonyl butoxide (PBO) were distributed in the 2010 and 2013 campaigns [8].

Insecticide resistance is defined as the ability of mosquitoes to survive exposure to a standard discriminating dose of insecticide [9]. Inevitably, after many years of prolonged use of pyrethroid insecticides to control agricultural pests and disease vectors, malaria vectors with increasing levels of pyrethroid resistance have emerged, and this has impacted on the ability of LLINs to control these mosquito populations [10, 11]. The impact of pyrethroid resistance on malaria transmission in Africa is contested [12–16]. The sometimes contradictory findings may be partially explained by the varying intensities of resistance in the study sites; a recent meta-analysis of bioassay studies and experimental hut trials data [17] shows that the community protection provided by nets reduces rapidly as resistance emerges whereas personal protection is only lost when resistance reaches much higher levels.

Although insecticide-resistant *An. gambiae* (*sensu stricto*), by definition, are not killed upon immediate contact with insecticides, fitness costs incurred from exposure may indirectly reduce their disease transmission potential [18]. Delayed mortality post-LLIN exposure has been demonstrated in a previous laboratory trial on pyrethroid-resistant colonies [19], and in a field study using *An. funestus* (*sensu lato*) and *An. gambiae* (*s.l*.) from Cameroon [20]. These studies found that the magnitude of the delayed mortality effects decreases in strains that have developed multiple resistance mechanisms and/or compensatory mutations [19, 20]. Given the rapid increase in resistance intensity observed in Burkina Faso and the emergence of additional potent resistance mechanisms [21, 22] we sought to quantify the presence of any delayed mortality following LLIN exposure in these highly resistant populations.

## Methods

### Study sites

Laboratory bioassays were performed in the insectaries at the Liverpool School of Tropical Medicine (LSTM), UK, and the Centre National de Recherche et de Formation sur le Paludisme (CNRFP) clinical research unit of Banfora, Burkina Faso (10°37′N, 04°46′W). Experimental hut studies were carried out at the CNRFP field station in Tengrela (10°40′N, 04°50′W). The huts are located on the outskirts of Tengrela village adjacent to rice growing fields. Tengrela is a rural town, mainly known as a rice and vegetable growing area, located in the Comoé Province approximately 440 km south-west of Ouagadougou, the country’s capital, and 7 km from the province’s capital, Banfora. Yendere (10°12′N, 04°58′W) is also a rural town with no specific agricultural practice. Cotton is grown in the areas surrounding the town. It is also in Comoé Province approximately 60 km from Banfora. Both sites are in the same health district of Banfora. The climate in this area of the country is characterised with a rainy season from June to October and a dry season from November to May. The average temperature is 27.5 °C and average annual rainfall is 1080 mm. Field experiments were conducted between 2016 and 2018 during the rainy season.

### Mosquito strains

Two laboratory strains (VK7 2014, hereafter referred to as VK7, and Banfora) and two field populations, collected as larvae from Tengrela and Yendere, of insecticide-resistant *An. gambiae* (*s.l*.) from Burkina Faso were used. The insecticide-susceptible *An. gambiae* (*s.s*.) Kisumu reference strain [23] was used as a control in experiments conducted at LSTM, and to test the efficacy of netting used for tests in Burkina field studies. The Banfora laboratory strain was colonised from the Tengrela field site in 2015 and the VK7 strain from Valle du Kou, village no. 7 in 2014. Both are *An. coluzzii* colonies and have been maintained at LSTM under standard insectary conditions (27 ± 2 °C, 80 ± 10% relative humidity (RH) with a 12:12 h light:dark photoperiod). Field populations were collected as larvae from Tengrela and Yendere over several collection days. Mosquitoes were sampled from different types of breeding site (e.g. temporary pools, rice fields). Larvae were reared to adults in the insectaries (25 ± 3 °C and 75 ± 25% RH) at CNRFP; these mosquitoes were used for insecticide bioassays and in reared-release studies in experimental huts. In Tengrela, mosquitoes were largely collected from rice fields. In Yendere, rice is not a major crop, and mosquitoes were collected from more temporary breeding sites, where typically *An. gambiae* (*s.s*.) predominate over *An. coluzzii* [24, 25]. Freely entering adults from Tengrela, of unknown age, were used in wild-entry experiments. Species identification of field strains was conducted using SINE PCR [26] at LSTM. *Anopheles coluzzii* predominates in Tengrela (87%, 437 mosquitoes tested) and *An. gambiae* (*s.s.*) in Yendere (90%, 203 mosquitoes tested).

### Insecticide resistance status

The VK7 and Banfora laboratory strains are resistant to permethrin, deltamethrin and DDT [27]. Topical and tarsal permethrin dose-response assays suggest the Banfora strain to be more pyrethroid-resistant than VK7 although this difference is not significant. VK7 has a high frequency of the 1014F kdr mutation with the 1575Y sodium channel mutation present at a low level; several P450s (CYP6M2, CYP6P3 and CYP6P4) with known pyrethroid metabolism activity are upregulated in this strain. The Banfora strain is also heterozygous for the 1014F and 1575Y sodium channel mutations; metabolic resistance is less predominant in this strain and instead, topical assays suggest insecticide penetration barriers contribute to the resistance phenotype [27]. To establish the resistance status of larval-reared field populations, WHO susceptibility tube bioassays [9] were performed using control and deltamethrin papers at the diagnostic dose (0.05%), plus further assays using papers of increasing deltamethrin concentrations (0.05%, 0.25%, 0.50%, 0.75% and 1.0%); daily survival following exposure was assessed. Details of sample sizes are provided in Additional file 1: Figure S1.

### Net treatments

PermaNet®2.0 (Vestergaard Frandsen, Switzerland, deltamethrin 1.4–1.8 g/kg) and untreated nets (purchased locally) were used for both LSTM laboratory tests and all field tests. Nets were aired for a minimum of one week prior to experiments (with the exception of the 2016 hut trials where nets were used on the same day, without airing) and acclimatised to the respective testing room before use. Details of sample sizes are provided in Additional file 1: Table S2.

### WHO cone bioassay

Mosquitoes were exposed to randomly selected pieces of untreated or PermaNet 2.0 netting using a standard three-minute WHO cone bioassay [28]. For laboratory assays and 2017 field tests, one untreated net and one PermaNet 2.0 were used for all tests. For field assays, in 2017 two untreated nets and two PermaNet 2.0 nets were used. Netting pieces were randomly sampled from the roof and sides of the nets. Cohorts were exposed to nets once (Assay A) or several times (Assays B–E) using a variety of differing test regimes (Table 1). For laboratory assays, cohorts of 70 mosquitoes were exposed, and for field assays cohorts ranged from 25–125 mosquitoes depending on availably of mosquitoes (details of sample sizes are provided in Additional file 1: Table S2). The laboratory and field assays were carried out at different times and locations. The different exposure regimes approximate alternative types of exposure to LLINs that mosquitoes may experience during their lifespan [19]. Assay A (single exposure) provided a baseline level of net contact to compare untreated and treated netting. Assays B, C, and E (daily exposure for 2, 3 and 5 days, respectively) simulates the net contact a mosquito might encounter if it is repeatedly prevented from obtaining a blood meal. Assay D (exposure every 4 days for 4 exposures) simulates the level of net contact a mosquito might encounter every gonotrophic cycle. The exposure regimes varied between the laboratory and field experiments for logistical reasons.

**Table 1:** Summary of experimental factors in cone bioassays. Mosquitoes were exposed to PermaNet 2.0 and untreated nets

Cone assay ID	LLIN exposure (times exposed)	Exposure regime	Mosquito strain	Age (days)^a^
A	Single (×1)	Exposed once	VK7 (Lab)	4
			Banfora (Lab)	4
			Yendere (Field)	3–5
			Tengrela (Field)	5–8
B	Multiple (×2)	Daily exposure for 2 consecutive days	VK7 (Lab)	4
			Banfora (Lab)	4
C	Multiple (×3)	Daily exposure for 3 consecutive days	VK7 (Lab)	4
			Banfora (Lab)	4
D	Multiple (×4)	Exposure every 4 days, for a maximum of 4 exposures	Tengrela (Field)	4
E	Multiple (×5)	Daily exposure for 5 consecutive days	Tengrela (Field)	4

^a^Age at first exposure

*Abbreviation*: Lab, laboratory

Age at first exposure to insecticides varied between 3 to 8 days post-eclosion and only non-blood-fed females were used. Mortality at 24 hours post-exposure was recorded. After the final exposure, all surviving mosquitoes were held with access to a sugar solution and daily mortality was recorded until all mosquitoes were dead.

### Experimental hut trials

The semi-field experimental hut station contained six huts built to the West African design [28] and is situated adjacent to Tengrela’s rice fields. Two trials (A and B) were conducted using either larval-reared mosquitoes or wild-entry mosquitoes, respectively over a two-year period (Table 2). Trials were replicated in 2016 and 2017. In Hut Trial B only mosquitoes without a visible bloodmeal were used to score longevity. Huts contained either untreated net (control) or unwashed PermaNet^®^ 2.0. Nets were holed according to WHO guidelines [28]. Sleepers were randomly rotated within huts; however, small mosquito numbers for release meant this occurred on non-consecutive days, and between two and six huts were used for trials (full details Additional file 1: Table S1).

**Table 2:** Summary of experimental factors in experimental hut trials

Trial	Mosquito population (strain)	Year conducted	Date conducted	No nights	Age (days)	Blood-feeding status
A	Reared-release (larval-reared Tengrela)	2016	26 September–3 October	6	5–8	Unfed; blood-fed
		2017	10–22 September	10	5–8	Unfed; blood-fed
B	Wild-entry (Tengrela)	2016	10–21 October	10	Unknown	No visible blood meal
		2017	2–15 July	12	Unknown	No visible blood meal

Volunteers entered the huts after ~20:00 h and remained under the nets until ~6:00 h. In the reared-release trial, window shutters, entries and door frames were closed or covered with untreated netting to prevent the exit of released mosquitoes. In the wild entry trial, window entries remained open. After acclimatisation (> 10 min) mosquitoes were either manually released into the hut (reared-release trial) or window traps opened to allow wild mosquitoes to enter (wild-entry trial).

The following morning, mosquitoes were collected individually using glass universal tubes and placed into labelled bags separated by location (i.e. under net, in the veranda, in the main hut). The remaining mosquitoes were collected using a Prokopack aspirator (The John W. Hock Company, Florida, USA). All mosquitoes were morphologically identified [29], sexed, recorded as dead or alive, and scored for abdominal status (unfed, partially-fed, blood-fed, semi-gravid/gravid). Dead female *Anopheles* mosquitoes were stored in silica, and male *Anopheles* and non-anopheline mosquitoes were recorded and discarded. Surviving female mosquitoes were transferred to paper cups and provided with 10% glucose solution. Mortality was recorded daily until all mosquitoes were dead, and dead mosquitoes were stored in silica.

### Data analysis

Chi-square or Fisherʼs exact test was used for immediate mortality analysis. If a mosquito was censored (e.g. mosquito escaped) during the 24 hours following exposure, it was removed from immediate mortality analysis. In discrimination dose bioassays, immediate mortality following insecticide exposure was always less than 5 % so Abbotʼs correction [9] was not applied. In cone bioassays following single exposure control mortality was low across all treatments (< 5%). As control mortality during subsequent exposures in multiple exposure assays could be affected by mosquito age, cone bioassay mortality was not corrected in any exposures. For survival analysis, Kaplan-Meier curves were used to visualise the data, and Cox regression was used to compare post-exposure survival. Immediate mortality (24-h post-exposure, and/or dead on collection) was excluded, and censored data included. All analysis was conducted in IBM SPSS Statistics 24 (IBM Corp. IBM SPSS Statistics for Windows, Version 24.0. Armonk, NY, USA).

A Bayesian state-space survival model as developed by Viana et al. [19] was used to quantify the daily survival rate and the magnitude of any observed delayed mortality effect in each experiment. Briefly, the observed number of mosquitoes alive each day was modelled from a binomial distribution described by the total number of mosquitoes alive and the probability of daily survival which, in turn was described with a logit link to its nonlinear predictor parameterised as a function of the treatment previously published [19]. The results were generated using this model executed in JAGS. The model, structure and parameter priors have been previously published elsewhere [19]. The results were generated using a version of the model executed using Mathcad.

## Results

### WHO cone bioassays

#### Immediate mortality

**Fig. 1:**
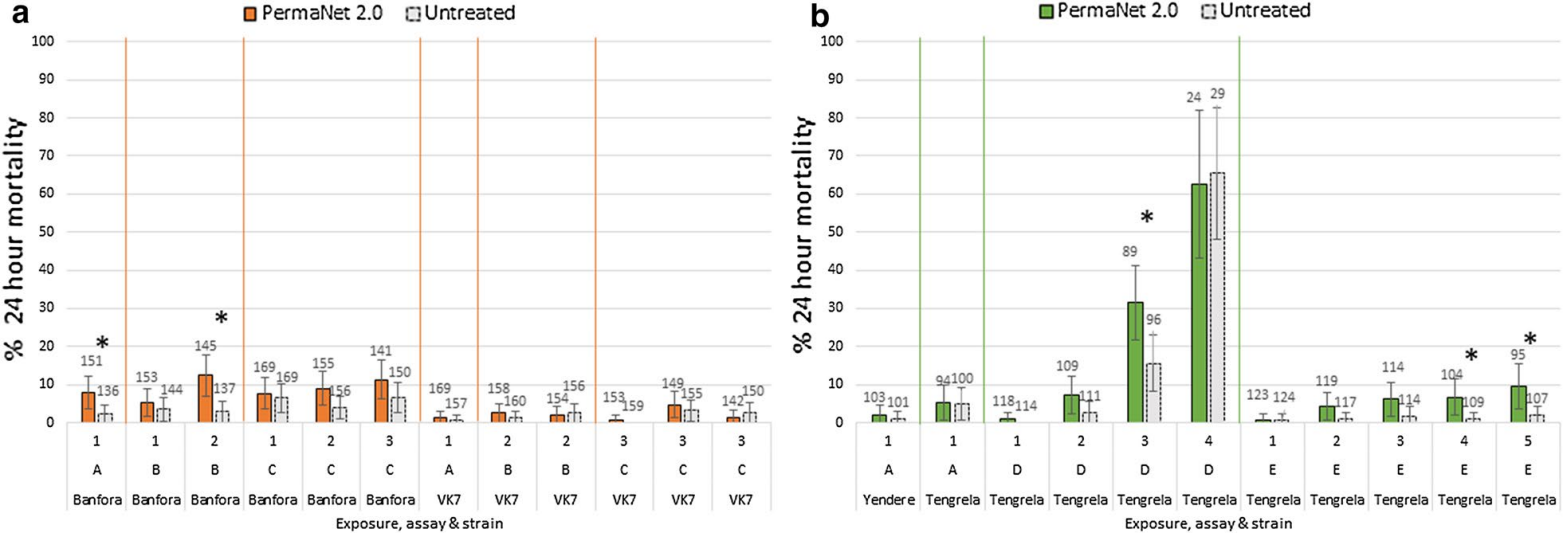
24-hour mortality of laboratory Banfora and VK7 (**a**) and field Tengrela and Yendere (**b**) mosquitoes after WHO cone bioassay exposure. Mosquitoes were exposed to PermaNet 2.0 or untreated net single (Assay A), or multiple (Assays B-E) times and their mortality recorded. Error bars show 95% confidence intervals for the population proportion. Numbers above bars show the number of mosquitoes tested. Numbers below the graph show the number of exposures and letters refer to the experimental design (see Table 1). Asterisks show when untreated and PermaNet 2.0 mortality was significantly different (*P* < 0.05). See Additional file 1: Table S2 for details of the mortality in each assay

The Kisumu susceptible strain showed high immediate mortality against PermaNet 2.0 (LSTM strain, 100% mortality, *n *= 100 mosquitoes; CNRFP strain, 98% mortality, *n *= 48 mosquitoes). In laboratory strains, after single and repeated exposure to PermaNet 2.0, the immediate mortality of the Banfora and VK7 was < 15% (Fig. 1a; Additional file 1: Table S2). In the laboratory strains, a significant difference between PermaNet 2.0 mortality and untreated net mortality was only seen in the Banfora strain, following the single exposure (Assay A, Fig. 1a, *P *= 0.029), and the second exposure of the two exposure assay (Assay B, Fig. 1a, *P *= 0.003). In all other exposures, no significant difference in immediate mortality between laboratory mosquitoes exposed to treated or untreated net was seen (Fig. 1a; Additional file 1: Table S2).

In the field strains (Tengrela and Yendere) no difference in immediate mortality between PermaNet 2.0 and the untreated net was observed following single exposures (Assay A). However, significantly higher mortality was observed after the third exposure in Assay D (4 exposures every four days), and the 4th and 5th exposure in Assay E (5 exposures daily) (Fig. 1b; Additional file 1: Table S2).

#### Delayed effects

**Fig. 2:**
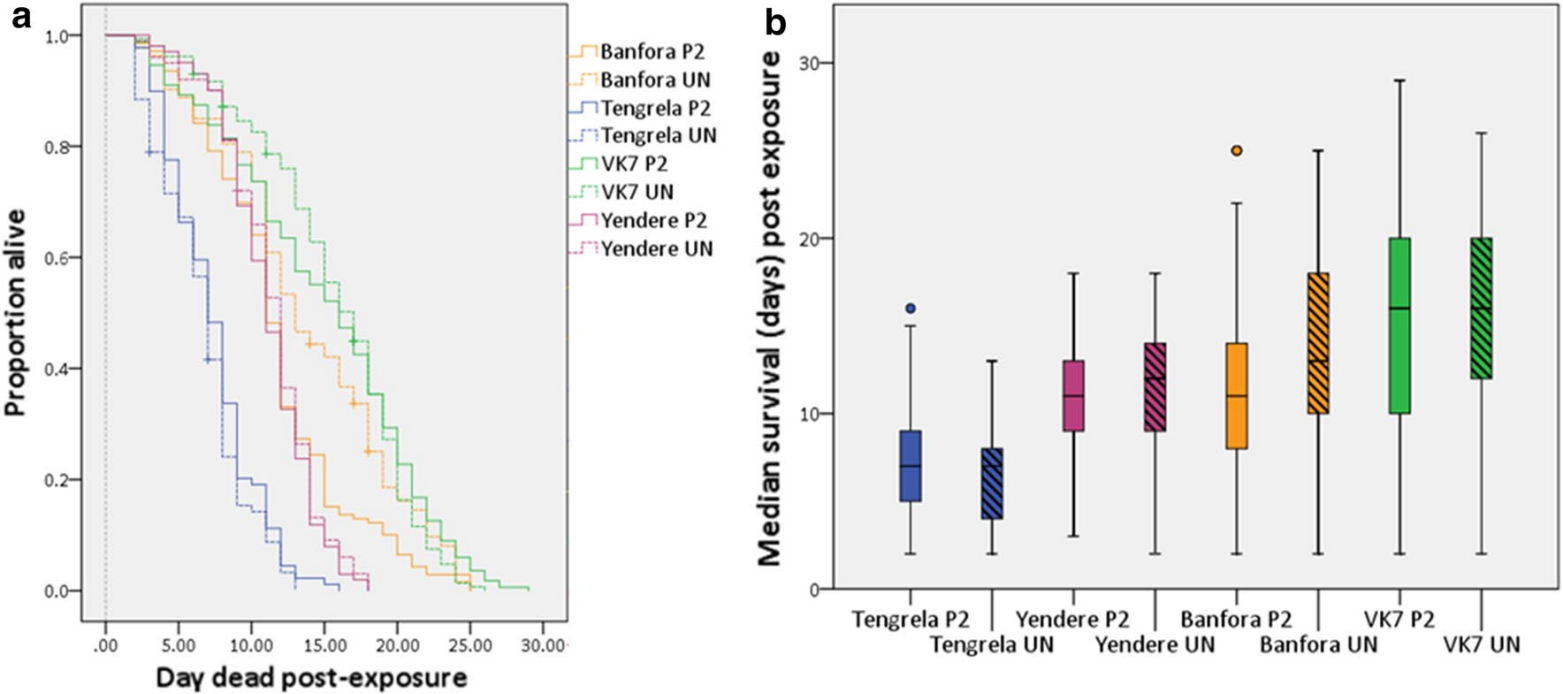
The longevity of laboratory and field populations after single WHO cone bioassay exposures. **a** Kaplan Meier survival curves show the proportion alive each day post-exposure. The dashed grey line indicates the day mosquitoes were exposed. Crosses represent censored data at the point of censoring. **b** Box and whisker plots of median survival (days) dead post-exposure. Mosquitoes were 4 (VK7 and Banfora), 3–5 (Yendere), or 5–8 (Tengrela) days-old on exposure. Coloured dots show outliers in the data. In both **a** and **b** immediate (within 24 h) mortality is excluded. Banfora: 2 replicates (PN2, *n *= 139 mosquitoes; UN, *n *= 133 mosquitoes); VK7: 2 replicates (PN2, *n *= 167 mosquitoes; UN, *n *= 156 mosquitoes); Tengrela: 2 replicates (PN2, *n *= 89 mosquitoes; UN, *n *= 95 mosquitoes); Yendere: 2 replicates (PN2, *n *= 101 mosquitoes; UN, *n *= 100 mosquitoes)

After a single exposure to LLINs, there was no significant reduction in survival compared to a single exposure to untreated netting in the laboratory VK7 strain (Cox regression, *P *= 0.57), and field Tengrela (Cox regression, *P *= 0.27) and Yendere (Cox regression, *P *= 0.52) populations (Fig. 2a). Only the laboratory Banfora strain showed significantly reduced survival after a single exposure to LLIN compared to the control (Cox regression, *P *= 0.03); Banfora mosquitoes exposed to PermaNet 2.0 had a 1.44-fold (95% CI: 1.13–1.84) increase in the risk of death compared to Banfora mosquitoes exposed to untreated netting.

**Fig. 3:**
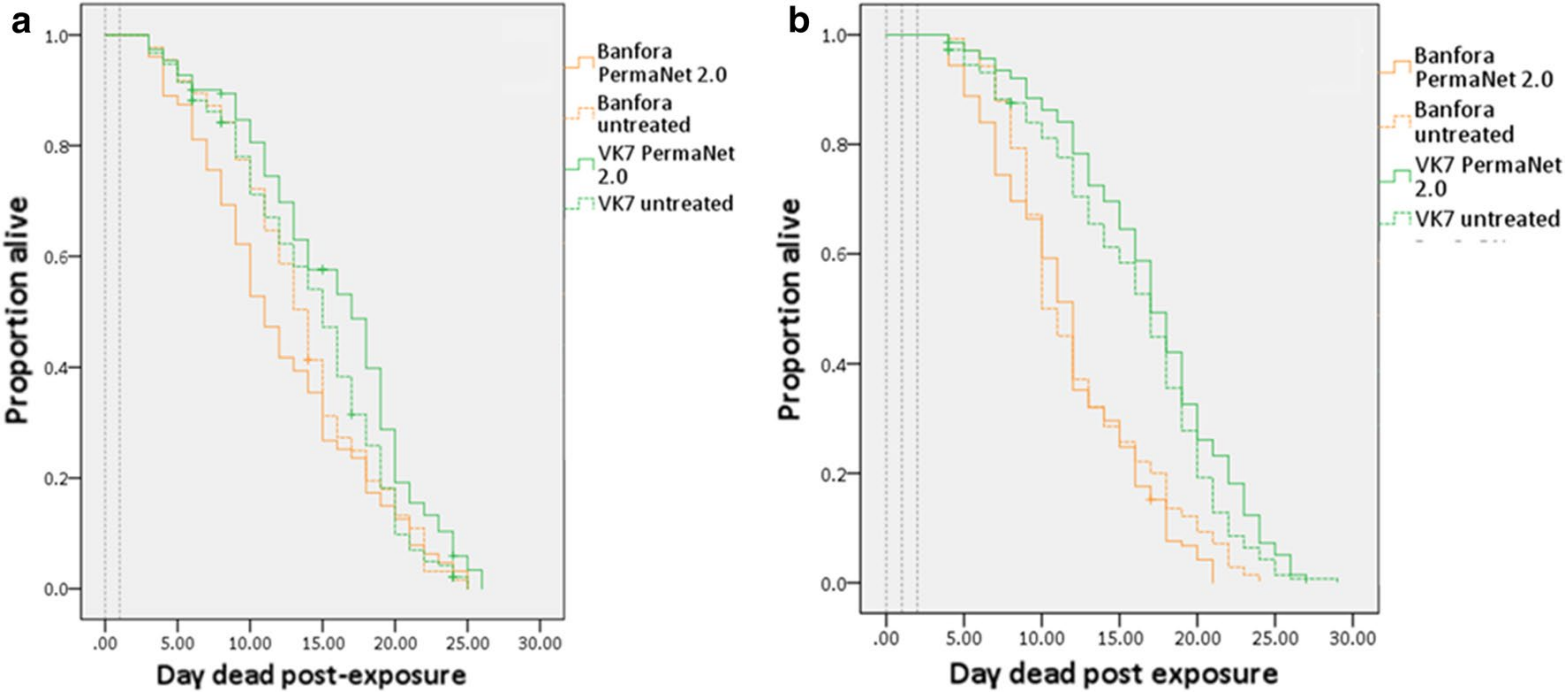
The longevity of laboratory strains after multiple WHO cone bioassay exposures. Kaplan Meier survival curves show the proportion alive each day following two (**a**) or three (**b**) exposures. The dashed grey line indicates the day mosquitoes were exposed. Crosses represent censored data at the point of censoring. In both **a** and **b** immediate (within 24 h) mortality is excluded

After two exposures to LLIN (Fig. 3a), the Banfora strain showed no significant reduction in cumulative survival compared to two exposures to untreated netting (Cox regression, *P *= 0.26), whilst the VK7 strain showed a small, but significant (Cox regression, *P *= 0.008) increase in survival after two exposures to LLIN compared to the control;VK7 exposed to PermaNet 2.0 had a 0.72-fold (95% CI: 0.57–0.92) decrease in the risk of death compared to controls. After three exposures (Fig. 3b) neither laboratory strain showed a reduction in longevity compared to untreated netting (Banfora, *P *= 0.206; VK7, *P *= 0.085).

**Fig. 4:**
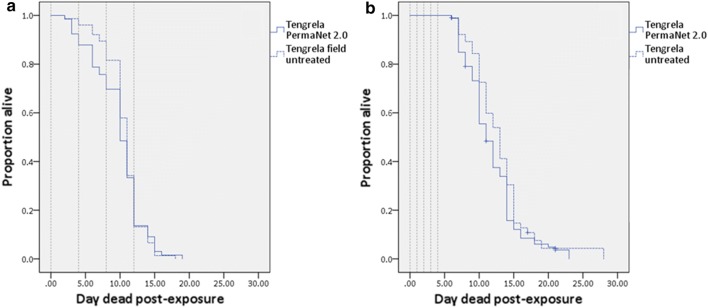
The longevity of field populations after multiple WHO cone bioassay exposures. Kaplan Meier survival curves show the proportion alive each day following four exposures every four days (**a**); or five daily exposures (**b**). In both **a** and **b** the dashed grey line indicates the day mosquitoes were exposed. Crosses represent censored data at the point of censoring. Immediate (within 24 h) mortality is excluded

The Tengrela field population was exposed to LLINs either every fourth day, four times (Assay D) or daily for five days (Assay E). Neither exposure regime had any impact on long-term survival compared to untreated netting [Fig. 4a (*P *= 0.72) and 4b (*P *= 0.97)].

### Experimental hut trials

#### Mosquito numbers, species identification and immediate mortality

Over the two-year study, a total of 1187 *Anopheles* and 602 non-*Anopheles* were collected during 22 nights by volunteer sleepers in the wild-entry experimental hut trials in Tengrela (Additional file 1: Table S1, Table S6). The average number of female *Anopheles* caught per night/per hut were 16.9 in 2016 and 6.00 in 2017 for PermaNet 2.0 huts, and 20.6 in 2016 and 8.08 in 2017 in untreated hut (Additional file 1: Table S6). Lower mosquito numbers in 2017 may be due to the trial being conducted early in the rainy season (July), whereas mosquito numbers in 2016 (October) are comparable to other hut trials conducted at this site [30]. In the release-recapture hut trials, 782 *Anopheles* were released and 493 recaptured across all huts. A total of 92 non-target (non-*Anopheles* or male *Anopheles*) were collected. Recapture rates were greater in untreated compared to PermaNet 2.0 huts over the two years (Additional file 1: Table S6; Untreated: 76.21%; PermaNet 2.0: 49.87). Molecular ID confirmed *An. coluzzii* to be the dominant species of mosquitoes collected from Tengrela (87.41% *An. coluzzii*; 2.97% *An. gambiae* (*s.s.*); 1.14% *An. coluzzii/gambiae* hybrids; 0.23% *An. arabiensis*; 8.24% unidentified; 437 mosquitoes tested in 2017), while *An. gambiae* (*s.s*.) were more abundant in mosquitoes collected from Yendere (90.15% *An. gambiae* (*s.s*.); 0.49% *An. coluzzii/gambiae* hybrids; 0.49% *An. arabiensis*; 6.40% unidentified; 203 mosquitoes tested in 2018).

In the reared-release trials, where adult mosquitoes aged 5 to 8 days, raised from larval collections were released into the huts, immediate mosquito mortality (dead on collection or within 24-h) in PermaNet 2.0 huts was 50% (95% CI: 38.61–61.39%) in 2016, and 45.50% (95% CI: 33.66–51.34%) in 2017 (untreated hut mortality: 2016, 11.01%, 95% CI: 5.13–16.89%; 2017, 16.22%, 95% CI: 10.90–21.53%). In the wild-entry trials, where mosquitoes were of unknown age, mortality in PermaNet 2.0 huts was 8.38% (95% CI: 4.18–12.59%) in 2016, and 13.57% (95% CI: 7.90–19.24%) in 2017 (untreated hut mortality: 2016, 4.93%, 95% CI: 1.95–7.90%; 2017, 5.29%, 95% CI: 2.10–8.48%). Mortality in the PermaNet 2.0 huts was always higher than in the huts with untreated nets but this difference was not significant in the wild entry trials. Further details of mosquito exophily and blood-feeding are provided in Additional file 1: Table S7.

#### Delayed mortality

**Fig. 5:**
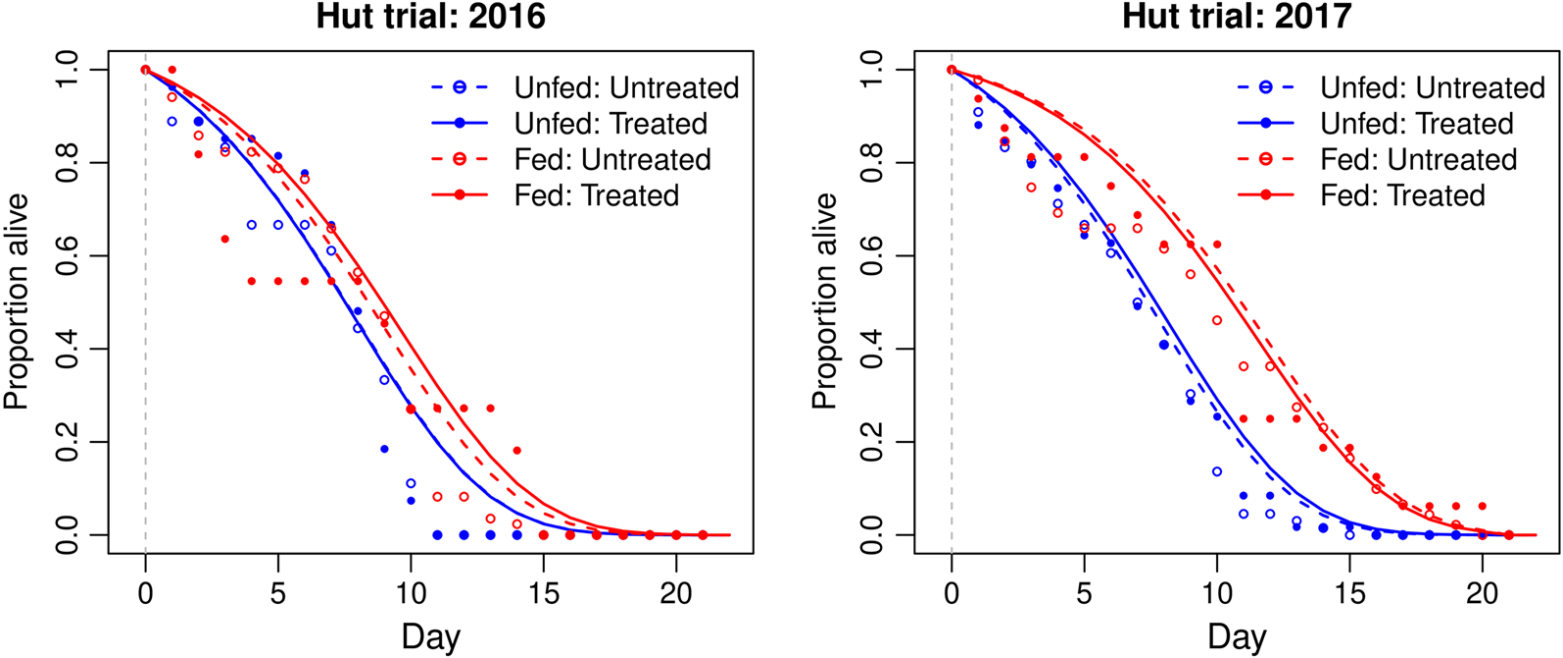
The longevity of field populations after exposure in reared-release hut trial. Daily survival curves from the state-space model show the proportion alive each day following collections of blood-fed and unfed mosquitoes in 2016 and 2017. Dashed grey lines represent day of insecticide exposure in the hut trial

The effect of date, feeding status, hut, net treatment, and collection locations (e.g. in net, in veranda) on mosquito survival post-collection was analysed. For the reared-released trials, in 2016, only blood-feeding status significantly affected mosquito longevity (Fig. 5, 92 blood-fed mosquitoes, 42 unfed mosquitoes, *P *= 0.001). When non-significant variables were excluded from the regression analysis, blood-fed mosquitoes had a 0.561-fold (0.384–0.819) lower risk of death (*P *= 0.003). In 2017, date of collection (*P *= 0.005) and blood-feeding status (*P* < 0.0001) both significantly affected mosquito longevity. When non-significant variables were removed from the model, and results were stratified by day, blood-fed mosquitoes had a 0.450-fold (0.327–0.618) reduction in the risk of death compared to unfed mosquitoes (Fig. 5b, 107 blood-fed mosquitoes, 113 unfed mosquitoes, *P* < 0.0001). Data were hence stratified into unfed and blood-fed groups. In the reared-release trials, exposure to LLINs had no effect on longevity in either 2016 or 2017 (Fig. 5, Additional file 1: Table S4).

**Fig. 6:**
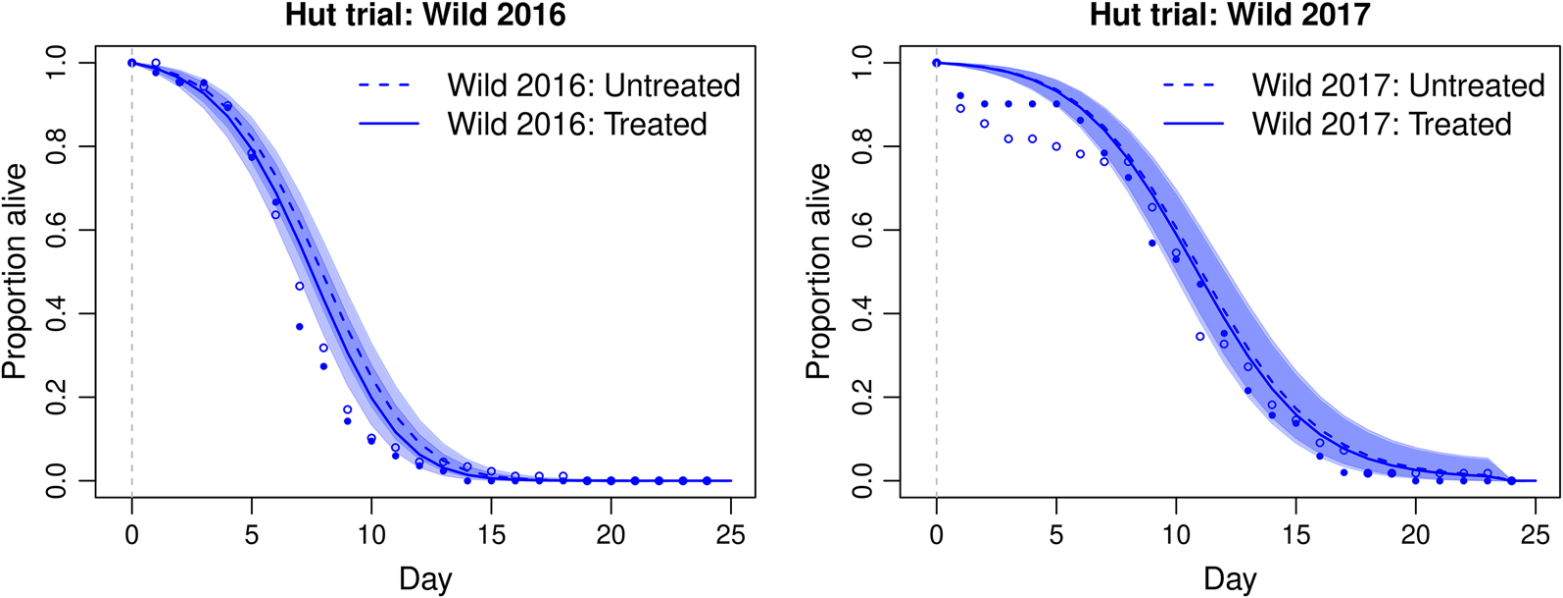
The longevity of field populations after exposure in wild-entry hut trials. Daily survival curves from the state-space model show the proportion alive each day following collections of blood-fed and unfed mosquitoes in 2016 and 2017. Dashed grey lines represent day of insecticide exposure in the hut trial. Shading represents 95% confidence intervals

In the wild-entry trials, only unfed mosquitoes were retained for post-collection longevity analysis (as blood-fed mosquitoes were used in a separate experiment to investigate reproductive output not presented here). Again, in these trials, net treatment had no significant effect on mosquito longevity (Fig. 6) in either 2016 (untreated hut, *n *= 85 mosquitoes; PermaNet 2.0 hut, *n *= 85 mosquitoes, *P *= 0.405) or 2017 (untreated hut, *n *= 55 mosquitoes; PermaNet 2.0 hut, *n *= 53 mosquitoes, *P *= 0.892).

### WHO intensity assays

In the discriminating dose assays, following exposure to the standard diagnostic dose of deltamethrin (0.05%), mortality was 2.01% for Tengrela (95% CI: -0.24–4.37%, *n *= 149 mosquitoes). As the insecticide concentration was increased to 5× and 10× the diagnostic dose, mortality increased but it then plateaued or even decreased at 15× and 20× concentrations possibly indicating that the solubility limit of deltamethrin had been exceeded at these higher concentrations; a significant difference between treated and control mortality was seen following exposure to 0.25%, 0.5%, 0.75% and 1% deltamethrin papers (Additional file 1: Figure S1).

**Fig. 7:**
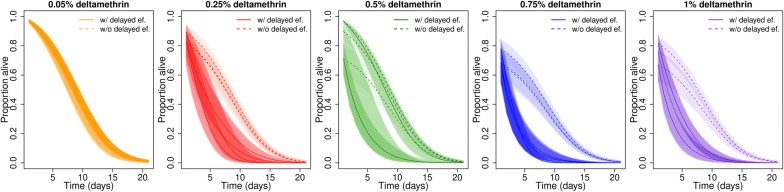
The modeled daily survival curves of *An. gambiae* following a WHO longevity tube assay. Mosquitoes from Tengrela were exposed to increasing concentrations of deltamethrin or untreated control papers. Full lines represent the curve estimated from fitting the binomial model to the data, and the dotted lines represent the counterfactual curve predicted with no delayed effects. Lines correspond to the median prediction with shaded 95% credible intervals

Excluding immediate mortality, there was no evidence of delayed mortality compared to untreated control at the standard dose of deltamethrin (0.05%, *P *= 0.395). However, as mosquitoes were exposed to increasing insecticide concentration, reduced longevity was observed in the treated versus the control tubes (Fig. 7; Additional file 1: Table S5).

## Discussion

In our earlier publication [19], we showed that exposure to LLINs resulted in a delayed mortality effect that approximately halved the overall mosquito lifespan beyond the 24 hours post-exposure. The magnitude of this delayed mortality varied between strains, with LLIN exposure having a greater impact on median mortality in a moderately resistant Tororo laboratory strain than in the more highly resistant Tiassalé strain. However, the potential impact on malaria transmission of this delayed mortality was substantial for both strains, with exposure to LLINs estimated to reduce the malaria transmission by 3.3-fold and 7.8-fold in Tororo and Tiassalé, respectively. At the time of publication, we noted that although this delayed mortality effect may be mitigating the impact of pyrethroid resistance on LLIN efficacy in the field, this effect may be eroded as resistance increases in intensity. We also recognised the importance of testing for delayed mortality in field populations, using more realistic methods of LLIN exposure. As a consequence, we have been routinely measuring daily survival post-insecticide exposure in our laboratory and field assessments of pyrethroid resistance. Here, we report data on the impact of LLIN exposure on lifelong survival in populations of *An. gambiae* (*s.l*.) from Burkina Faso.

Southwestern Burkina Faso is known as a hotspot for pyrethroid resistance [30]. We established two colonies of *An. coluzzii* from this region at LSTM in 2014 (VK7) and 2015 (Banfora), both of which have higher levels of pyrethroid resistance than our previous ‘gold standard’ resistant strain, Tiassalé [27]. Multiple exposures to LLINs in cone bioassays had very little impact on the 24-hour post-exposure with mortality levels less than 12% in all cases. Furthermore, there was no evidence of any delayed mortality in any of the exposure regimes for the VK7 strain. Delayed mortality was only observed in the Banfora strain although the magnitude of this effect was much smaller than observed in previous studies with Tiassalé and Tororo colonies (< 6% reduction in daily mortality in Banfora due to delayed mortality effects *vs* 46% for Tororo and 12% for Tiassalé).

When cone bioassays were performed directly on mosquitoes collected from the field, again there was very little immediate mortality following LLIN exposure and no evidence of any delayed mortality. The 3-minute exposure used in the cone bioassays is a simple means of evaluating the response in the laboratory but does not reflect the realities of mosquito exposure to LLINs in the field. Indeed, the duration of contact of mosquitoes with LLINs in response to a human baited bed net has been shown to be less than three minutes [31]. The use of experimental huts enabled us to mimic LLIN exposure in the field under controlled conditions. Again, we observed no difference between the longevity of mosquitoes exposed to LLINs or control nets.

In hut trials, feeding status had a significant effect on mosquito longevity with blood-fed mosquitoes surviving significantly longer post-collection than unfed mosquitoes. During blood meal digestion mosquitoes upregulate enzymes to detoxify harmful products from the blood meal. Subsequently, these enzymes could be providing an additional benefit following exposure to insecticides by assisting in insecticide detoxification [32]. In other laboratory trials acquiring a blood meal has been shown to improve survival following insecticide exposure [33] and increase longevity [34] and similar effects have documented in other field locations [35].

Reared released mosquitoes (Hut trial A, Fig. 6a, b), did not survive as long post-exposure as the wild entry mosquitoes in hut trial B (Fig. 7a, b). The experimental huts in Tengrela are situated between the rice fields and the village, and it is anticipated that a large proportion of mosquitoes in the wild entry experiments may be newly eclosed mosquitoes seeking their first blood meal. Females used in the reared release trials were five to eight days-old. The presumed difference in age structure between the wild mosquitoes entering the experimental huts and the reared and released, may explain the differences in observed longevity as it is well documented that mosquito susceptibility to insecticides increases as they age [36–38]. Additionally, by collecting and rearing mosquitoes in the insectary for release, we may be including mosquitoes of lower fitness which in the wild may have died before reaching the huts. Additionally, the extra handling and transportation of the larval-reared mosquitoes to the hut station in the reared-release trial may have led to increased mortality, although we note that only a slight increase is observed in the untreated arm of the reared-release trial, in comparison to the wild-entry trial suggesting this may have a relatively minor impact on the differential mortality observed in the two tests.

Having observed almost no impact of LLIN exposure on mosquito longevity in any of the populations or exposure regimes, we sought to understand whether delayed mortality could be induced by increasing the amount of insecticide the mosquitoes were exposed to. Here we found that there was evidence of a delayed mortality effect at concentrations of > 5× the discriminating dose in WHO tubes assay. These results indicate pyrethroids can induce sub-lethal effects even in the highly resistant populations, but under standard exposure conditions, these effects are rarely evident.

## Conclusions

Mosquito longevity is the primary determinant of vectorial capacity. Our findings that standard pyrethroid nets are not impacting on the longevity of malaria vectors in southwestern Burkina Faso are of great concern. This study did not measure other potential sub-lethal effects of pyrethroid exposure in the resistant populations, such as reproductive output or re-feeding success, and these are now being investigated in follow-up studies. Further studies on the impact of exposure of pyrethroid-resistant mosquito populations on *Plasmodium* development are also needed to fully understand the impact of resistance on malaria transmission potential.

## Supplementary information

**Additional file 1: : Table S1.** Summary of the number of nights volunteer and net treatment spent in each hut during trials. **Table S2.** Summary of 24-hour mortality from WHO cone bioassay exposures. **Table S3.** Estimated and counterfactual mean daily mosquito survival after WHO cone bioassay exposure. **Table S4.** Estimated and counterfactual mean daily mosquito survival after exposure in the reared-release trial. **Table S5.** Estimated and counterfactual mean daily mosquito survival after exposure in WHO tube assay. **Table S6.** Summary of mosquitoes in release-recapture and wild entry hut trials. In reared-release trials percentages show *Anopheles* recapture rate. **Table S7.** Summary of outcomes of *An. gambiae* s.l. in wild-entry hut trial in 2016 and 2017. **Figure S1.** The 24 hr mortality of An. gambiae s.l from Tengrela (2018) following exposure to deltamethrin diagnostic dose (0.05%) and intensity (0.10, 0.25, 0.50, 0.75, 1.00%) doses or an untreated control, in WHO tube bioassays. **Figure S2.** The longevity of laboratory populations after exposure in WHO cone assays.

## Data Availability

Data supporting the conclusions of this article are included within the article and its additional file. The datasets used and/or analysed during the present study are available from the corresponding author upon reasonable request.

## Abbreviations

CNRFP: Centre National de Recherche et de Formation sur le Paludisme
LLIN: long-lasting insecticidal nets
LSTM: Liverpool School of Tropical Medicine
PBO: piperonyl butoxide
PPF: pyriproxyfen
WHO: World Health Organization
