# Supplementary material for: Anopheles gambiae populations from Burkina Faso show minimal delayed mortality after exposure to insecticide-treated nets
Source: Parasit Vectors. 2020 Jan 10;13:17. doi: 10.1186/s13071-019-3872-2 (PMC6954553; doi:10.1186/s13071-019-3872-2)
Supplement: Supplementary file 1 — Additional file 1: : Table S1. Summary of the number of nights volunteer and net treatment spent in each hut during trials. Table S2. Summary of 24-hour mortality from WHO cone bioassay exposures. Table S3. Estimated and counterfactual mean daily mosquito survival after WHO cone bioassay exposure. Table S4. Estimated and counterfactual mean daily mosquito survival after exposure in the reared-release trial. Table S5. Estimated and counterfactual mean daily mosquito survival after exposure in WHO tube assay. Table S6. Summary of mosquitoes in release-recapture and wild entry hut trials. In reared-release trials percentages show Anopheles recapture rate. Table S7. Summary of outcomes of An. gambiae s.l. in wild-entry hut trial in 2016 and 2017. Figure S1. The 24 hr mortality of An. gambiae s.l from Tengrela (2018) following exposure to deltamethrin diagnostic dose (0.05%) and intensity (0.10, 0.25, 0.50, 0.75, 1.00%) doses or an untreated control, in WHO tube bioassays. Figure S2. The longevity of laboratory populations after exposure in WHO cone assays. [file 13071_2019_3872_MOESM1_ESM.docx]

**Additional file 1**

**Table S1.** Summary of the number of nights volunteer and net treatment spent in each hut during trials.

| **Year** | **Trial** | **Volunteer/net treatment** | **Hut number** | | | | | |
| --- | --- | --- | --- | --- | --- | --- | --- | --- |
|  |  |  | **1** | **2** | **3** | **4** | **5** | **6** |
| 2016 | Reared-release | Sleeper SH |  |  |  |  | 4 | 2 |
|  |  | Sleeper OY |  |  |  |  | 2 | 4 |
|  |  | Untreated Net |  |  |  |  | 5 | 1 |
|  |  | PermaNet 2.0 |  |  |  |  | 1 | 5 |
|  | Wild-entry | Sleeper SH |  |  |  |  | 6 | 4 |
|  |  | Sleeper OY |  |  |  |  | 4 | 6 |
|  |  | Untreated Net |  |  |  |  | 5 | 5 |
|  |  | PermaNet 2.0 |  |  |  |  | 5 | 5 |
| 2017 | Reared-release | Sleeper SH |  | 4 | 2 |  |  |  |
|  |  | Sleeper OY |  | 2 | 4 |  |  |  |
|  |  | Untreated Net |  | 5 | 1 |  |  |  |
|  |  | PermaNet 2.0 |  | 1 | 5 |  |  |  |
|  | Wild-entry | Sleeper BA | 1 | 2 | 1 | 1 | 2 | 1 |
|  |  | Sleeper SO | 1 | 2 | 1 | 1 | 2 | 1 |
|  |  | Sleeper SA | 1 | 2 | 1 | 1 | 2 | 1 |
|  |  | Sleeper IS | 1 | 2 | 1 | 1 | 2 | 1 |
|  |  | Sleeper YA | 1 | 2 | 1 | 1 | 2 | 1 |
|  |  | Sleeper MO | 1 | 2 | 1 | 1 | 2 | 1 |
|  |  | Untreated Net | 6 | 6 |  | 6 | 6 |  |
|  |  | PermaNet 2.0 |  | 6 | 6 |  | 6 | 6 |

Abbreviations: SH, OY, BA, SO SA IS, YA MO (anonymised volunteer names)

**Table S2.** Summary of 24-hour mortality from WHO cone bioassay exposures.

| **Strain/**  **population** | **Cone assay ID** | **Exposure** | **Net** | **Total**  **mosquitoes** | **% Mortality with 95% CI** | | | **P-value** |
| --- | --- | --- | --- | --- | --- | --- | --- | --- |
|  |  |  |  |  | **Lower** | **%** | **Upper** |  |
| Banfora | A | 1 | P2 | 151 | 3.63 | 7.95 | 12.26 | 0.029 |
|  |  |  | Un | 136 | -0.26 | 2.21 | 4.67 |  |
|  | B | 1 | P2 | 153 | 1.70 | 5.23 | 8.76 | 0.460 |
|  |  |  | Un | 144 | 0.48 | 3.47 | 6.46 |  |
|  |  | 2 | P2 | 145 | 7.05 | 12.41 | 17.78 | 0.003 |
|  |  |  | Un | 137 | 0.10 | 2.92 | 5.74 |  |
|  | C | 1 | P2 | 169 | 3.67 | 7.69 | 11.71 | 0.672 |
|  |  |  | Un | 169 | 2.79 | 6.51 | 10.23 |  |
|  |  | 2 | P2 | 155 | 4.52 | 9.03 | 13.54 | 0.062 |
|  |  |  | Un | 156 | 0.83 | 3.85 | 6.86 |  |
|  |  | 3 | P2 | 141 | 6.11 | 11.35 | 16.58 | 0.162 |
|  |  |  | Un | 150 | 2.67 | 6.67 | 10.66 |  |
| VK7 | A | 1 | P2 | 169 | -0.45 | 1.18 | 2.81 | 1.000 |
|  |  |  | Un | 157 | -0.61 | 0.64 | 1.88 |  |
|  | B | 1 | P2 | 158 | 0.08 | 2.53 | 4.98 | 0.446 |
|  |  |  | Un | 160 | -0.47 | 1.25 | 2.97 |  |
|  |  | 2 | P2 | 154 | -0.23 | 1.95 | 4.13 | 1.000 |
|  |  |  | Un | 156 | 0.08 | 2.56 | 5.04 |  |
|  | C | 1 | P2 | 153 | -0.62 | 0.65 | 1.93 | 0.490 |
|  |  |  | Un | 159 | 0.00 | 0.00 | 0.00 |  |
|  |  | 2 | P2 | 149 | 1.30 | 4.70 | 8.10 | 0.510 |
|  |  |  | Un | 155 | 0.44 | 3.23 | 6.01 |  |
|  |  | 3 | P2 | 142 | -0.53 | 1.41 | 3.35 | 0.685 |
|  |  |  | Un | 150 | 0.09 | 2.67 | 5.24 |  |
| Yendere | A | 1 | P2 | 103 | 4.61 | 1.94 | -0.72 | 1.000 |
|  |  |  | Un | 101 | 2.92 | 0.99 | -0.94 |  |
| Tengrela | A | 1 | P2 | 94 | 9.86 | 5.32 | 0.78 | 1.000 |
|  |  |  | Un | 100 | 9.27 | 5.00 | 0.73 |  |
|  | D | 1 | P2 | 118 | 2.50 | 0.85 | -0.81 | 1.000 |
|  |  |  | Un | 114 | 0.00 | 0.00 | 0.00 |  |
|  |  | 2 | P2 | 109 | 12.24 | 7.34 | 2.44 | 0.115 |
|  |  |  | Un | 111 | 5.72 | 2.70 | -0.31 |  |
|  |  | 3 | P2 | 89 | 41.11 | 31.46 | 21.81 | 0.011 |
|  |  |  | Un | 96 | 22.89 | 15.63 | 8.36 |  |
|  |  | 4 | P2 | 24 | 81.87 | 62.50 | 43.13 | 0.820 |
|  |  |  | Un | 29 | 82.82 | 65.52 | 48.22 |  |
|  | E | 1 | P2 | 123 | 2.40 | 0.81 | -0.77 | 1.000 |
|  |  |  | Un | 124 | 2.38 | 0.81 | -0.77 |  |
|  |  | 2 | P2 | 119 | 7.81 | 4.20 | 0.60 | 0.213 |
|  |  |  | Un | 117 | 2.52 | 0.85 | -0.81 |  |
|  |  | 3 | P2 | 114 | 10.55 | 6.14 | 1.73 | 0.171 |
|  |  |  | Un | 114 | 4.16 | 1.75 | -0.66 |  |
|  |  | 4 | P2 | 104 | 11.55 | 6.73 | 1.92 | 0.032 |
|  |  |  | Un | 109 | 2.71 | 0.92 | -0.87 |  |
|  |  | 5 | P2 | 95 | 15.36 | 9.47 | 3.58 | 0.017 |
|  |  |  | Un | 107 | 4.44 | 1.87 | -0.70 |  |

**Table S3.** Estimated and counterfactual mean daily mosquito survival after WHO cone bioassay exposure.

| **Strain** | **Number of exposures** | **Mean daily survival** | |
| --- | --- | --- | --- |
|  |  | **Estimate** | **Counterfactual**  **(without delayed effects)** |
| Banfora | Control 1 | 0.81 | - |
|  | 1 | 0.76 | 0.79 |
|  | Control 2 | 0.79 | - |
|  | 2 | 0.78 | 0.82 |
|  | Control 3 | 0.74 | - |
|  | 3 | 0.75 | 0.82 |
| VK7 | Control 1 | 0.83 | - |
|  | 1 | 0.84 | 0.84 |
|  | Control 2 | 0.80 | - |
|  | 2 | 0.83 | 0.83 |
|  | Control 3 | 0.83 | - |
|  | 3 | 0.84 | 0.85 |
| Tengrela | Control 1 | 0.47 | - |
|  | 1 | 0.51 | 0.55 |
|  | Control 4 | 0.66 | - |
|  | 4 | 0.63 | 0.63 |
|  | Control 5 | 0.75 | - |
|  | 5 | 0.67 | 0.68 |
| Yendere | Control 1 | 0.70 | - |
|  | 1 | 0.69 | 0.69 |

**Table S4.** Estimated and counterfactual mean daily mosquito survival after exposure in the reared-release trial.

| **Year** | **Status** | **Number of exposures** | **Mean daily survival** | |
| --- | --- | --- | --- | --- |
|  |  |  | **Estimate** | **Counterfactual**  **(without delayed effects)** |
| 2016 | Unfed | Control | 0.68 | - |
|  |  | 1 | 0.68 | 0.68 |
|  | Fed | Control | 0.74 | - |
|  |  | 1 | 0.74 | 0.74 |
| 2017 | Unfed | Control | 0.69 | - |
|  |  | 1 | 0.69 | 0.69 |
|  | Fed | Control | 0.81 | - |
|  |  | 1 | 0.80 | 0.80 |

**Table S5.** Estimated and counterfactual mean daily mosquito survival after exposure in WHO tube assay.

| **Deltamethrin**  **concentration** | **Replicate date** | **Reduction in survival 24h post-exp*** | **Mean daily survival** | |
| --- | --- | --- | --- | --- |
|  |  |  | **Estimate** | **Counterfactual**  **(w/out delayed effects)** |
| Control | 06 | - | 0.81 | - |
|  | 09 | - | 0.81 | - |
|  | 16 | - | 0.81 | - |
|  | 22 | - | 0.81 | - |
|  | 30 | - | 0.81 | - |
| 0.005 | 06 | 1.0 | 0.80 | 0.81 |
|  | 09 | 1.0 | 0.80 | 0.81 |
|  | 16 | 1.0 | 0.81 | 0.81 |
|  | 22 | 1.0 | 0.81 | 0.81 |
|  | 30 | 1.0 | 0.81 | 0.81 |
| 0.025 | 06 | 2.2 | 0.75 | 0.80 |
|  | 08 | 2.1 | 0.50 | 0.80 |
|  | 09 | 1.5 | 0.55 | 0.80 |
| 0.05 | 06 | 3.3 | 0.67 | 0.79 |
|  | 08 | 1.0 | 0.77 | 0.80 |
|  | 09 | 1.5 | 0.54 | 0.80 |
| 0.75 | 06 | 3.9 | 0.65 | 0.79 |
|  | 08 | 3.7 | 0.67 | 0.79 |
|  | 09 | 2.6 | 0.36 | 0.80 |
| 1 | 08 | 3.9 | 0.65 | 0.79 |
|  | 09 | 2.2 | 0.68 | 0.80 |

*Rate of reduction in the mean daily survival of the exposed strain in the 24 h following insecticide exposure compared to unexposed control.

**Table S6.** Summary of all mosquitoes in release-recapture and wild entry hut trials. In reared-release trials percentages show *Anopheles* recapture rate.

| Trial ID |  | 2016 | | 2017 | |
| --- | --- | --- | --- | --- | --- |
|  |  | Untreated net | PermaNet 2.0 | Untreated net | PermaNet 2.0 |
| Reared- release | Female *Anopheles*  (recapture rate) | 113 (78.47%) | 75 (52.45%) | 185 (74.90%) | 120 (48.39%) |
|  | Male *Anopheles* & non-*Anopheles* | 12 | 46 | 17 | 17 |
| Wild-entry | Female *Anopheles* (per night/ per hut) | 206 (20.6) | 169 (16.9) | 194 (8.08) | 144 (6.00) |
|  | Male *Anopheles* & non-*Anopheles* | 309 | 248 | 284 | 235 |

**Table S7.** Summary of outcomes of *An. gambiae* s.l. in wild-entry hut trial in 2016 and 2017.

|  | **2016** | | **2017** | |
| --- | --- | --- | --- | --- |
|  | **Untreated net** | **PermaNet 2.0** | **Untreated net** | **PermaNet 2.0** |
| Total *An. gambaie* s.l collected | 206 | 169 | 194 | 144 |
| % deterrence | - | 17.96 | - | 25.77 |
| % exophily | 27.18  (21.11 – 33.36) | 28.40  (21.60 – 35.20) | 15.46  (10.38 – 20.55) | 16.67  (10.58 – 22.75) |
| Induced exophily | **-** | 4.29 | - | 7.22 |
| % blood-fed | 54.85  (48.06 – 61.65) | 46.15  (38.64 – 53.67) | 69.07  (62.57 – 75.58) | 52.78  (44.62 – 60.93) |
| % feeding inhibition | **-** | 15.86 | - | 23.59 |
| % mortality | 4.93  (1.95 – 7.90) | 8.38  (4.18 – 12.59) | 5.29  (2.10 – 8.48) | 13.57  (7.90 – 19.24) |
| % personal protection | **-** | 30.97 | - | 43.28 |
| % killing effect | **-** | 1.97 | - | 4.76 |

*Deterrence: the reduction in hut entry of mosquitoes in treatment huts relative to untreated huts; Exophily: mosquitoes found in veranda as a proportion of the total number collected in the hut; Induced exophily: the increase in mosquitoes in the veranda in treatment huts compared to untreated huts; Blood-feeding inhibition: the reduction in blood-feeding mosquitoes in treatment huts compared to untreated huts. Mortality: mosquitoes found dead on collection or dying within the first 24-hours as a proportion of those collected. Personal protection: 100 ×((Blood fed mosquitoes in untreated hut-Blood fed mosquitoes in treated hut))/(Blood fed mosquitoes in untreated hut); Killing effect: 100 ×((Mosquitoes killed in treated nets-Mosquitoes killed in untreated nets))/(Total mosquitoes collected in untreated hut)*


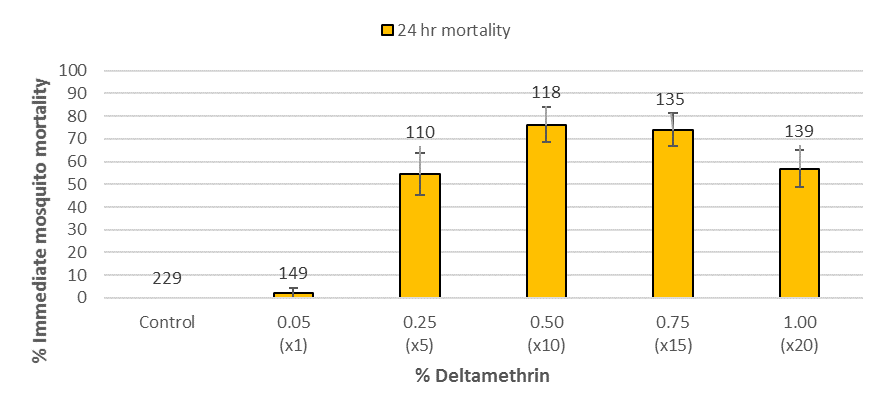


**Figure S1. The 24hr mortality of *An*. *gambiae* s.l from Tengrela (2018) following exposure to deltamethrin diagnostic dose (0.05%) and intensity (0.10, 0.25, 0.50, 0.75, 1.00%) doses or an untreated control, in WHO tube bioassays.** Error bars show 95% confidence intervals for the population proportion. Numbers above bars show the number of mosquitoes tested. 0.25% deltamethrin, 54.55% mortality, n = 110, p = 0.000; 0.50% deltamethrin, 76.27% mortality, n = 118, p = 0.000; 0.75% deltamethrin, 74.07% mortality, n = 135, p = 0.000; 1.00% deltamethrin, 56% mortality, n = 129, p = 0.000.


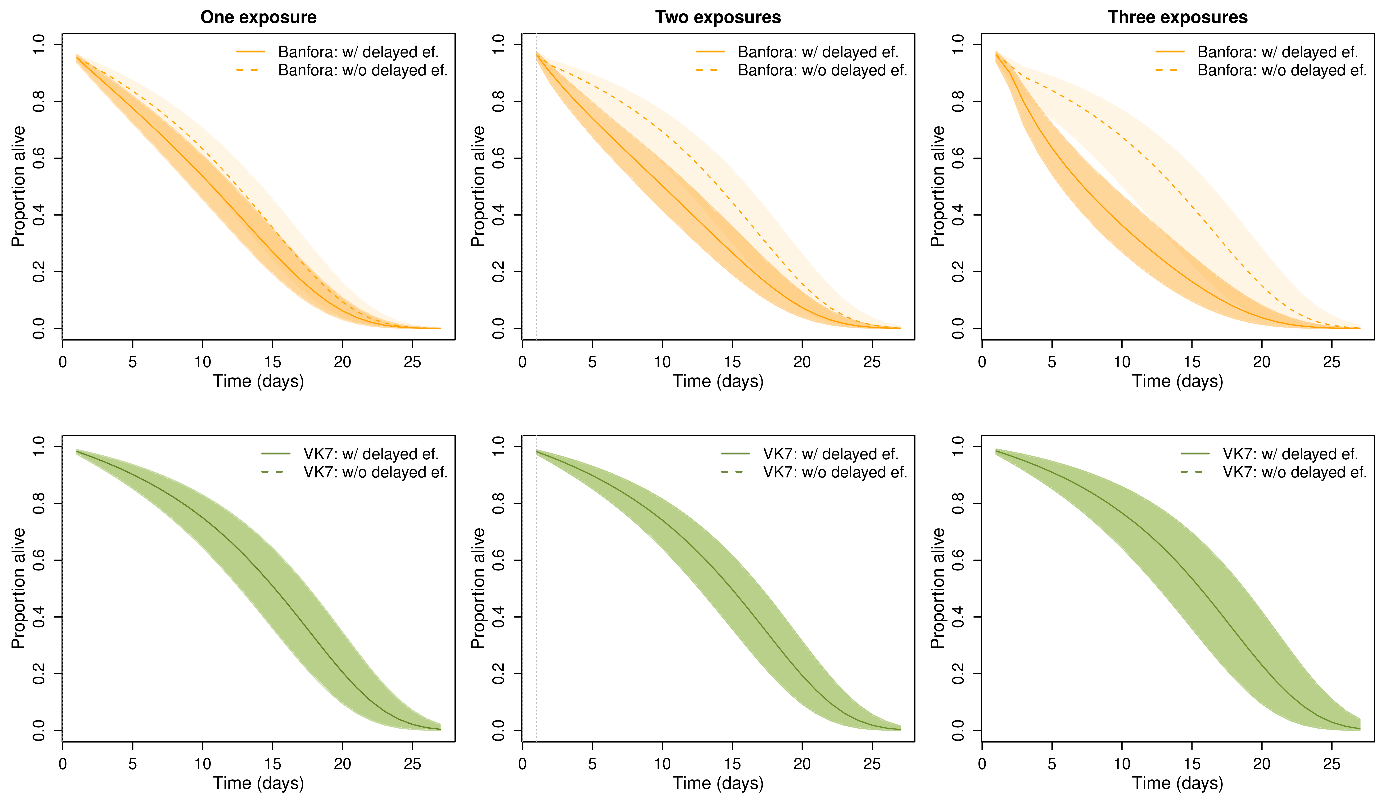


**Figure S2. The longevity of laboratory populations after exposure in WHO cone assays.** Daily survival curves from the state-space model show the proportion alive each day following exposure. Shading represents 95% confidence intervals. This is an example showing evidence of delayed mortality effects.
